# Electroencephalographic Responses to the Number of Objects in Partially Occluded and Uncovered Scenes

**DOI:** 10.1162/jocn_a_02264

**Published:** 2025-01-02

**Authors:** Cemre Baykan, Alexander C. Schütz

## Abstract

Perceptual completion is ubiquitous when estimating properties such as the shape, size, or number of objects in partially occluded scenes. Behavioral experiments showed that the number of hidden objects is underestimated in partially occluded scenes compared with an estimation based on the density of visible objects and the amount of occlusion. It is still unknown at which processing level this (under)estimation of the number of hidden objects occurs. We studied this question using a passive viewing task in which observers viewed a game board that was initially partially occluded and later was uncovered to reveal its hidden parts. We simultaneously measured the electroencephalographic responses to the partially occluded board presentation and its uncovering. We hypothesized that if the underestimation is a result of early sensory processing, it would be observed in the activities of P1 and N1, whereas if it is because of higher level processes such as expectancy, it would be reflected in P3 activities. Our data showed that P1 amplitude increased with numerosity in both occluded and uncovered states, indicating a link between P1 and simple stimulus features. The N1 amplitude was highest when both the initially visible and uncovered areas of the board were completely filled with game pieces, suggesting that the N1 component is sensitive to the overall Gestalt. Finally, we observed that P3 activity was reduced when the density of game pieces in the uncovered parts matched the initially visible parts, implying a relationship between the P3 component and expectation mismatch. Overall, our results suggest that inferences about the number of hidden items are reflected in high-level processing.

## Introduction

When some parts of a scene are not directly visible because of occlusion or gaps in the sensory processing, our visual system completes the missing information (for reviews, see Komatsu, 2006; Pessoa, Thompson, & Noë, 1998). In the context of object perception, there are two well-known visual completion phenomena: modal and amodal completion (Kanizsa, 1979; Michotte, Thinès, & Crabbé, 1964). In modal completion, our brain forms subjective or illusory shapes despite the absence of physical contours (Petry & Meyer, 2012; Kanizsa, 1979). Amodal completion occurs when the covered parts of occluded shapes are perceptually completed by means of sensory information from their surroundings, including edges, T-junctions, and curvatures (Emmanouil & Ro, 2014; Rauschenberger, Liu, Slotnick, & Yantis, 2006; Sekuler, 1994). Although there is abundant evidence for the mechanisms of perceptual completion on the level of simple objects (Palmer, Brooks, & Lai, 2007; de Wit & van Lier, 2002; Kellman & Shipley, 1991), the underlying processes on the level of scenes are not fully understood.

Visual scenes are often defined as real-world environments consisting of semantically organized objects (Epstein, Higgins, & Thompson-Schill, 2005; Henderson & Hollingworth, 1999). The perception of a scene relies on the properties of the contained objects and their mutual positioning (Greene & Oliva, 2009). Our visual system compares and enriches the visual input with internal models or *“*scene schemata*”* that reflect prior information about similar scenes (Wang, Foxwell, Cichy, Pitcher, & Kaiser, 2024; Kaiser & Cichy, 2021; Torralba & Oliva, 2003). For example, when visual information in the scene is ambiguous (blurry) so that local information is not sufficient for recognition of a single object, observers are able to describe a scene with cars, buildings, and the sky depending on spatial arrangement of the objects (Oliva & Torralba, 2006). General statistical properties of a scene such as object density, along with semantic relationships, contribute to the overall scene perception (Geisler, 2008). Importantly, humans are quite good at estimating such summary statistics, for example, the average size, orientation, and center of a collection of objects (Whitney & Yamanashi Leib, 2018; Albrecht, Scholl, & Chun, 2012; Alvarez & Oliva, 2008; Ariely, 2001). It is likely that the contents of hidden parts of a scene are inferred based on the visible information, including the spatial arrangement and general statistical properties of the visible objects, as supported by behavioral and fMRI findings (Men, Altin, & Schütz, 2023; Muckli et al., 2015; Smith & Muckli, 2010). For instance, it was shown that when scenes are partially occluded, our visual system reconstructs the missing information in the sensory representation of the environment by receiving feedback information from higher cortical areas (Muckli et al., 2015; Smith & Muckli, 2010).

A complete representation of the visual environment requires inferences about hidden (occluded) parts. For example, when foraging for berries on a bush, the number of hidden berries might be as relevant as the number of directly visible ones. Numerosity is considered a primary visual attribute (Anobile, Cicchini, & Burr, 2016), and humans use numbers spontaneously to discriminate between different groups of objects (Cicchini, Anobile, & Burr, 2016). Nevertheless, they do not accurately estimate the number of hidden objects compared with a prediction of equal distribution and constant density: Although they perceive the density of visible objects and the amount of occlusion accurately, they underestimate the number of hidden objects (Men et al., 2023). This underestimation could be an outcome of early sensory processing or high-level processes such as metacognitive biases or expectancy. It is therefore an open question what the underlying mechanism of the perceptually completed representation of a scene is. Do observers perceive a small number of objects behind an occluder, or is their decision guided by their expectancy about the given partially occluded scene? Here, using EEG, we studied whether numerosity perception in partially occluded scenes is a result of early sensory processing of the scene and/or expectancy about it.

In this study, we investigated the underlying mechanisms behind the (under)estimation of the number of hidden objects. We used a passive viewing task in which participants viewed a game board that was first partially occluded before it was uncovered completely. By doing so, we could measure the electrophysiological responses to both viewing of a partially occluded scene and its uncovering. Our first prediction was that early visual activities in the EEG should be related to simple visual properties of the display. A number of EEG studies have identified early ERPs that are associated with sensory processing of incoming information such as P1 and N1 (Johannes, Münte, Heinze, & Mangun, 1995; Heinze et al., 1994). Previous studies found that the amplitude of the P1 component scales with visual properties such as luminance and brightness, whereas N1 and P2 scale with continuous sensory cues in a scene such as aggregate surface, density, or average diameter of an array of dots (Soltész & Szűcs, 2014; Gebuis & Reynvoet, 2013; Hyde & Spelke, 2009; Libertus, Woldorff, & Brannon, 2007; Proverbio & Zani, 2002; Johannes et al., 1995). In line with these findings, we expected that an increasing number of items revealed behind an occluder should induce increasing activities of early visual sensory components (P1, N1, and P2).

Our second prediction was that uncovered scenes that follow an overall Gestalt structure should elicit higher N1 amplitudes. Previous research found that the N1 component is sensitive to perceptual completion (Tivadar, Retsa, Turoman, Matusz, & Murray, 2018; Hazenberg & van Lier, 2016; Wang, Zhang, Wang, Cui, & Tian, 2003; Proverbio & Zani, 2002). For example, an enhanced N1 amplitude was found for completed illusory contours (e.g., Pac-man-shaped inducers resulting in a Kanizsa-type square perception) relative to conditions in which the same stimuli did not give rise to a perceptual completion (Altschuler et al., 2012, 2014; Murray et al., 2002; Proverbio & Zani, 2002).

Furthermore, if high-level processes, such as expectation or metacognitive biases, contribute to the underestimation of the number of hidden objects, we should observe a higher activity in late processing stages. Perceptual experiences are often influenced by high-level processes such as expectancy or beliefs about objects or scenes (Hazenberg & van Lier, 2016; Rauschenberger, Peterson, Mosca, & Bruno, 2004). In concrete scenes (e.g., room layouts), perception of object numerosity is influenced by the semantics of a scene, relying on prior information, whereas in scenes containing abstract objects such as geometrical figures (like in our experiment), the perception of quantity is more uncertain. In abstract scenes, numerosity estimation could be based on an assumption of equal distribution of objects, such that there is a constant density; for example, if half of a scene is occluded, one can expect the same number of objects behind the occluder as objects are visible. Uncovering a different number of objects behind the occluder then would lead to an expectation violation. The processes of outcome evaluation and/or expectation violation can be indexed by several ERPs such as P3, MMN, or feedback-related negativity (Näätänen, Kujala, & Light, 2019; Stefanics & Czigler, 2015; Soltész & Szűcs, 2014; Holroyd, Coles, & Nieuwenhuis, 2002; Friedman, Cycowicz, & Gaeta, 2001; Miltner, Braun, & Coles, 1997). For instance, in a similar study to ours, Hazenberg and van Lier (2016) used the P3 component as a marker of surprise and found that P3 amplitude is enhanced when uncovered partially occluded objects violate observers*’* expectation about those objects. Therefore, our third prediction was that there should be an enhanced activity of P3 component when individuals see an uncovered scene that is incompatible to their expectations about the partially occluded scene. More specifically, we hypothesized that there would be an enhanced P3 activity in the uncovered phase when the numerosity of hidden pieces did not match with constant density prediction.

## Methods

### Participants

Twenty-three individuals took part in the study. All participants signed informed consent forms before the experiment and were paid 10 € per hour for their participation. The sample size was determined based on the sample sizes of similar EEG studies exploring the neural mechanisms of perceptual completion or numerosity (Gebuis & Reynvoet, 2013; Altschuler et al., 2012; Conci, Töllner, Leszczynski, & Müller, 2011), in which 12–18 participants were recruited. We used a sample size of 23 to increase the statistical power of the study. We excluded from further analyses the data of two participants because of equipment malfunction and the data of one participant because of excessive artifacts. Therefore, we analyzed and reported the results of the data of 20 participants (12 female, 8 male, mean age of 21.7 years, aged between 19 and 44 years). All participants were naive to the purpose of the study. The study was approved by the ethics committee of the Psychology Department at the University of Marburg (Proposal Number 2021-71 k).

### Stimuli and Procedure

The visual scene consisted of a game board, game pieces, and a mesh as an occluder. The game board was a gray square of 6.01° × 6.01° (luminance of 75.84 cd/m^2^, R= G = B = 150). The game pieces were black (luminance of 1.67 cd/m^2^, R = G = B = 0) and white (luminance of 128.28 cd/m^2^, R = G = B = 255) circles of 0.53°diameter. The occluder mesh was an array of black-and-white pixels of 8.02° × 8.02°, with the pixels randomized in each trial. This occluder was chosen to prevent possible visual after-effects following the occluder removal. To facilitate the occlusion perception, a shading effect was applied as though light was coming from the top left corner. All stimuli were presented on a dark gray background (luminance of 14.49 cd/m^2^, R = G = B = 26) via MATLAB (MathWorks Inc.) using Psychtoolbox 3 (Kleiner, Brainard, & Pelli, 2007). Participants viewed the stimuli on a back-projection setup (120-Hz PROPixx projector with 1920 × 1080 resolution, VPixx Technologies, and a 91 × 51 cm Stewart Filmscreen) at a viewing distance of 106 cm, which was kept fixed with a chin rest.

**Figure 1:**
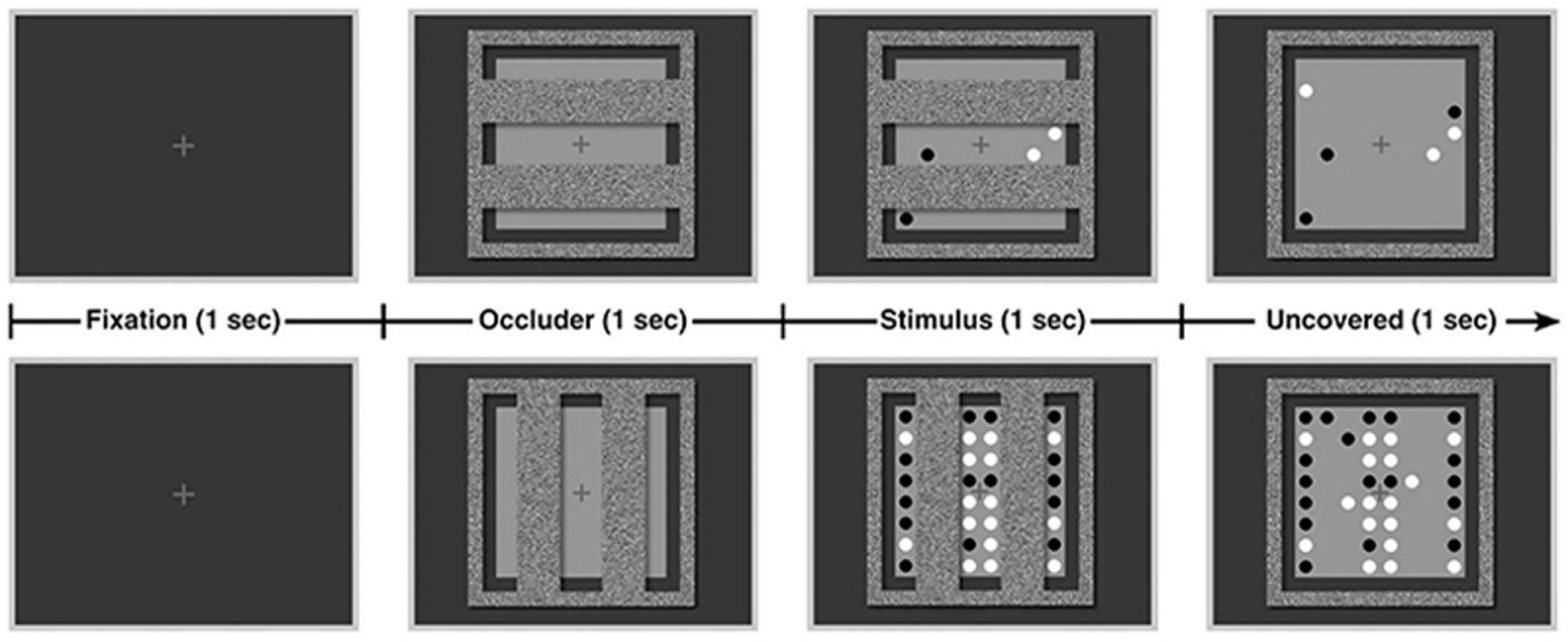
Illustration of a trial procedure. Participants performed a passive viewing task in which they viewed a stimulus display of a partly occluded board. Each trial started with a fixation cross for 1 sec plus the duration of the drift correction procedure. Following this, the occluder mesh with either horizontal or vertical stripes was shown for 1 sec. Next, 4 (upper row) or 32 (lower row) game pieces visible through the gaps of the occluder were presented for 1 sec. Finally, the occluder was removed to reveal the hidden parts of the board, uncovering 0–32 game pieces. The uncovered display was presented for another 1 sec.

Figure 1 illustrates a trial sequence. Participants performed a passive viewing task in a dimly lit room. Each trial started with a fixation cross (luminance of 51.06 cd/m^2^, R = G = B = 102) presentation for 1 sec plus the duration of the drift correction procedure. The game board and occluder were presented for 2 sec, and the game pieces only appeared in the last 1 sec of this presentation. Following the *“*partially occluded scene,*”* the occluder disappeared to uncover the hidden parts of the game board along with the visible game pieces leading to the *“*uncovered scene*”* phase. The uncovered scene was presented for 1 sec.

The experiment consisted of eight blocks of 80 trials each. There were two conditions of initially visible game pieces: 4 or 32 pieces, each with 8 uncovered conditions: 0, 1, 2, 4, 28, 30, 31 or 32 uncovered game pieces. All 16 conditions were repeated 40 times during the experiment, summing up to 640 trials in total. For 4 initially visible pieces, constant density would correspond to 4 additional pieces hidden from view, whereas 32 visible pieces would result in 32 additional pieces.

### Eye Tracking

We performed eye tracking using the Eyelink 1000 system (SR Research). A 9-point calibration procedure was performed twice: at the beginning of and halfway through the experiment. Each trial started with a custom-written automatic drift check procedure: After collecting 10 samples (gaze positions), they were averaged; if the average gaze position deviated >1.5° from the center of the screen, sample collection restarted; otherwise, drift check procedure was stopped. Sample collection restarted also if participants*’* current gaze position deviated >0.75° from the previous sample. Participants were instructed to fixate on the fixation cross throughout a trial. If they did not maintain their center fixation during a trial and their gaze deviated >1.5° from the center of the screen, they heard a *“*beep*”* sound after a trial ended.

### EEG Recording and Preprocessing

We recorded electrical brain activities from 64 scalp positions using the actiCAP Plus system and the BrainVision Recorder (Version 1.25.0202) software (Brain Products GmbH). The activity was amplified by a BrainAmp amplifier (DC to 280Hz) and sampled at the rate of 1000 Hz. Two electrodes were placed at the outer canthi of the right and left eyes for monitoring horizontal eye movements, and one electrode was placed below the left eye for monitoring vertical eye movements and eye blinks. We employed the MNE-Python (Version 1.0.3) package (Gramfort et al., 2013) for EEG data preprocessing. Whereas the electrode Fz was an online reference, the offline rereferencing used an average of the temporal–parietal electrodes (TP9 and TP10). The data were resampled to 500 Hz and bandpass-filtered from 0.1 to 40 Hz using a Hamming-windowed sinc finite impulse response filter. Artifacts caused by eye blinks, eye movements, and muscle noises were removed using an independent component analysis with the fastica algorithm.

### ERP Components

We examined the ERP activities both in the partially occluded and uncovered phases: the early visual ERP components—P1, N1, and P2—for the partially occluded phase and the activities of early visual ERP components—P1 and N1—and late visual ERP component P3 for the uncovered phase. We derived their activities from the symmetrical pairs of the parieto-occipital electrode cluster (PO8, PO4, PO3, and PO7), given that previous research commonly identify the largest activity for perceptual completion at this site of the scalp (Altschuler et al., 2012; Foxe, Murray, & Javitt, 2005; Murray et al., 2002). We segmented EEG data separately for the partially occluded and uncovered phases, from −0.2 to 1.0 sec relative to the presentation of the partially occluded and uncovered scene, respectively. For both phases, we baselined the data to the average voltage in the interval from −0.2 to 0 sec in the segments. We then averaged the signals per participant, per the number of initially visible pieces, and per the number of uncovered pieces. We calculated the mean P1 amplitude between 90 and 120 msec, the mean N1 amplitude between 140 and 170 msec, the mean P2 amplitude between 200 and 300 msec, and the mean P3 amplitude between 300 and 400 msec poststimulus onset, in line with the previous work (Hazenberg & van Lier, 2016; Gebuis & Reynvoet, 2013).

### Data Analysis

The ERP data underwent analysis using pairwise *t* tests via the *rstatix* (Version 0.7.2) package (Kassambara, 2023), repeated-measures ANOVA via the *ez* (Version 4.4–0) package (Lawrence, 2016), and linear models via the *stats* (Version 3.6.2; R Core Team, 2023) package in R (Version 4.3.2; R Core Team, 2023).

## Results

### Partially Occluded Phase

**Figure 2:**
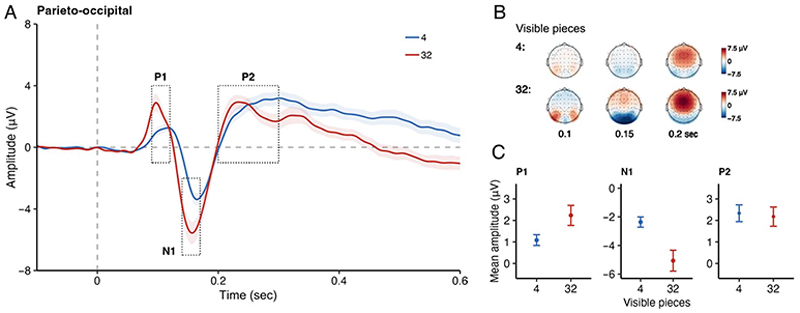
(A) Grand average ERPs over the parieto-occipital electrodes, relative to the occluded stimulus presentation. The shaded area is the standard error of the mean (*SEM*). Dotted rectangles depict the time window in which ERPs were calculated. (B) Topographies of P1, N1, and P2 amplitudes for 4 visible (upper row) and 32 visible (lower row) pieces. (C) Mean ERP amplitudes. Error bars are *SEM* (A and C). Blue represents 4 visible pieces and red 32 visible pieces.

Figure 2A illustrates the activity during the partially occluded phase over the parieto-occipital electrodes for different numbers of initially visible game pieces (4 or 32), and Figure 2B shows the topo-maps of these conditions at 0.1, 0.15, and 0.2 sec. In the partially occluded phase, we examined the mean amplitudes of P1, N1, and P2 components over the parieto-occipital scalp region by running separate pairwise *t* tests (Figure 2C). The results showed the mean P1 amplitude (± standard error) to be significantly larger for 32 (2.24 ± 0.47 μV) than for 4 visible pieces (1.09 ± 0.25 μV), *t*(19) = 3.23, *p* = .004, *d* = 0.72. Moreover, the mean N1 amplitude was also significantly larger for 32 (−5.06 ± 0.73 μV) than for 4 visible pieces (−2.36 ± 0.36 μV), *t*(19) = −5.46, *p* < .001, *d* = −1.22. A separate pairwise *t* test on the P2 mean amplitude showed that the activities of the P2 component were comparable for 32 (2.18 ± 0.45 μV) and 4 visible pieces (2.33 ± 0.39 μV), *t*(19) = −0.46, *p* = .65, *d* = −0.10.

### Uncovered Phase

**Figure 3:**
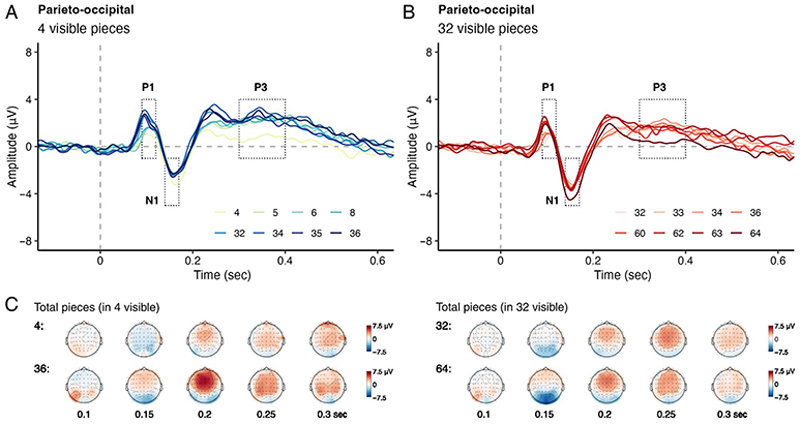
Grand average ERPs over the parieto-occipital electrodes, relative to the stimulus uncovering for (A) the 4 visible piece condition and (B) the 32 visible piece condition. Dotted rectangles depict the time windows in which ERPs were calculated in A and B. (C) Topographies of ERP amplitudes from 0.1 to 0.3 sec for 4 visible (left) and 32 visible (right) pieces.

**Figure 4:**
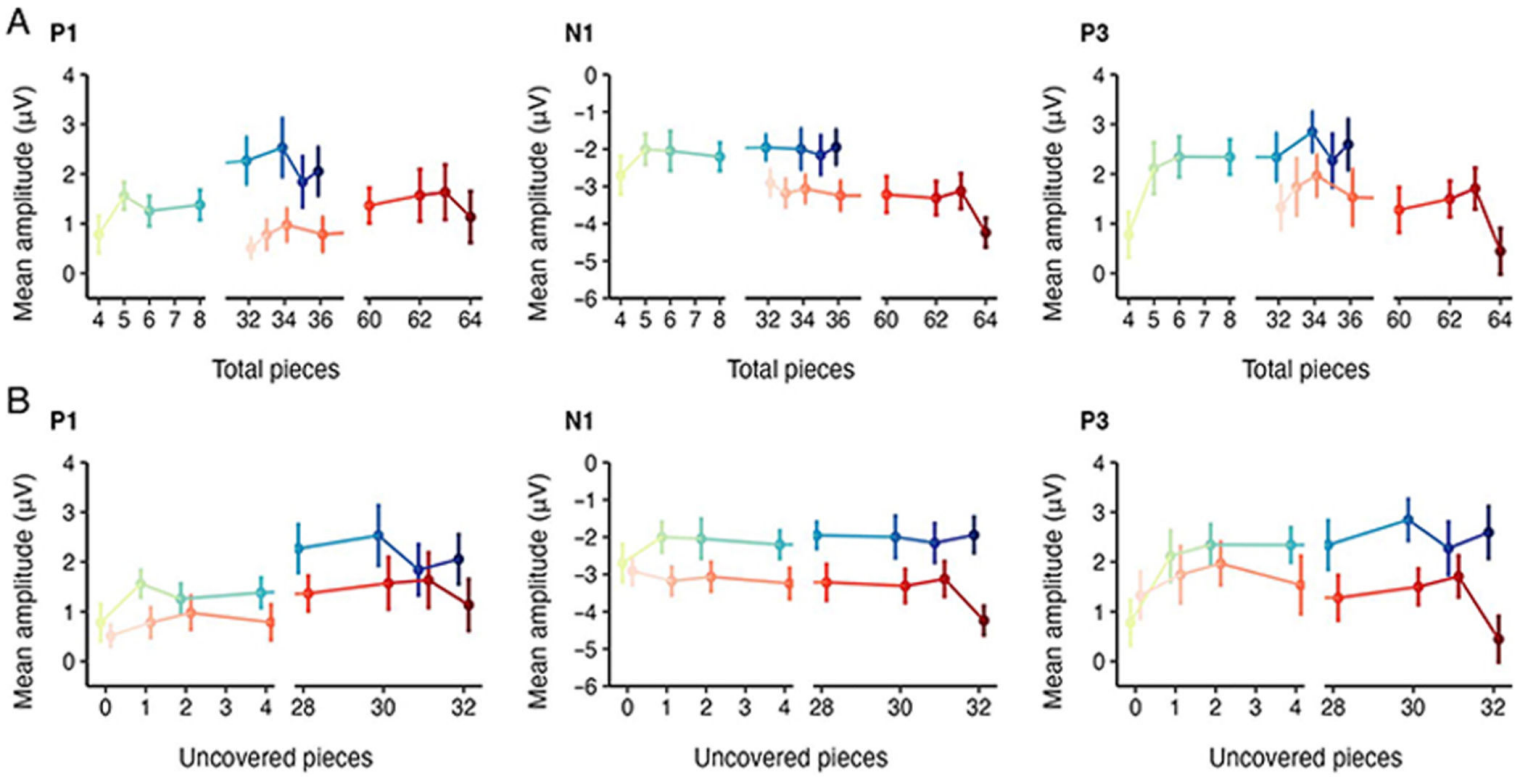
(A) Mean ERP amplitudes for the total game pieces visible on the board for P1 (left), N1 (middle), and P3 (right) components. (B) Mean ERP amplitudes for the uncovered game pieces behind the occluder for P1 (left), N1 (middle), and P3 (right) components. Error bars are *SEM*. Please note that the same ERPs are plotted in a different arrangement in A and B.

**Figure 5:**
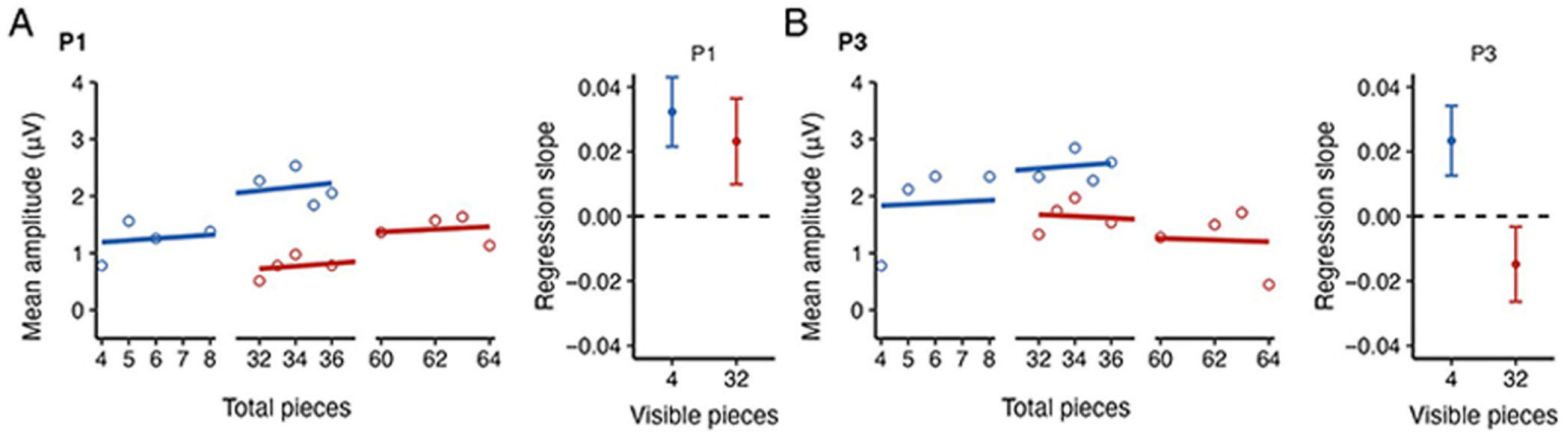
Blue represents 4 visible pieces, and red represents 32 visible pieces. (A; left) The estimated linear regression lines for the P1 component. Circles depict the averaged data. (Right) The estimated regression slopes across participants. (B; left) The estimated linear regression lines for the P3 component. Circles depict the averaged data. (Right) The estimated regression slopes across participants.

Figure 3 illustrates the activity during the uncovered phase over the parieto-occipital electrodes for different uncovered scenes presented after 4 (Figure 3A) and 32 initially visible pieces (Figure 3B). Figure 4A shows P1, N1, and P3 potentials plotted against the number of total game pieces. Figure 4B shows the same ERPs as Figure 4A, but as a function of the number of uncovered pieces. Given that P1 and N1 components were sensitive to the number of initially visible pieces, we further examined the mean amplitudes of these components over the parietooccipital region in the uncovered phase. In addition, we measured the activity of the P3 component. We examined the activities of these components by employing separate linear regression models with the number of initially visible pieces, hereafter *“*visible pieces,*”* as a categorical predictor (4 pieces as 0, 32 pieces as 1) and the number of total game pieces seen on the board after the occluder was removed, hereafter *“*total pieces*”* as a continuous predictor. Figure 5 depicts the estimated regression lines and slopes for P1 (Figure 5A) and P3 (Figure 5B) components.

#### P1

Figure 4A (left) illustrates the mean P1 amplitude during the uncovered phase for different uncovered scenes. The mean P1 amplitude was 1.71 (±0.33) μV for 4 visible pieces and 1.09 (±0.26) μV for 32 visible pieces. A multiple linear regression model on the P1 amplitude showed that there was a significant intercept (β = 1.06, 95% confidence interval [CI] [0.56, 1.56], *t*(316) = 4.19, *p* < .001). This indicates that the expected P1 amplitude for 4 visible pieces would have been 1.06 μV when the number of total game pieces on the board would have been zero. Furthermore, the results showed that the expected P1 amplitude for 32 visible pieces was comparable to 4 visible pieces (β = −1.08, CI [−2.21, 0.05], *t*(316) = −1.88, *p* = .061). There was a significant slope of the number of total pieces for 4 visible pieces (β = 0.03, CI [0.01, 0.05], *t*(316) = 3.14, *p* = .002), indicating 0.03-μV increase in the P1 amplitude, with each game piece seen in the board after the occluder was removed. The slope for 32 visible pieces did not differ significantly from 4 visible pieces (β = 0.01, CI [−0.04, 0.02], *t*(316) = −0.63, *p* = .53).

#### N1

Figure 4A (middle) illustrates the mean N1 amplitude during the uncovered scene phase for different uncovered scenes. The mean N1 amplitude was −2.13 (±0.38) μV for 4 visible pieces and −3.29 (±0.34) μV for 32 visible pieces. A separate multiple linear regression model on the N1 amplitude showed that there was a significant intercept (β = −2.29, CI [−2.82, −1.76], *t*(316) = −8.49, *p* < .001), which indicates that the expected N1 amplitude for 4 visible pieces would have been −2.29 μV when the number of total pieces would have been zero. The estimated N1 intercept for 32 visible pieces was comparable to 4 visible pieces (β = −0.31, CI [−1.51, 0.90], *t*(316) = −0.50, *p* = .62). There was no significant effect of total game pieces seen on the board for 4 visible pieces (β = 0.01, CI [−0.01, 0.03], *t*(316) = 0.74, *p* = .46). The slope for 32 visible pieces did not differ from the 4 visible pieces (β = −0.02, CI [−0.05, 0.01], *t*(316) = −1.45, *p* = .15).

These results showed that there was no linear relationship between the N1 amplitude and its predictor, the total game pieces. Therefore, we further analyzed the data using a two-way repeated-measures ANOVA on the N1 mean amplitude, with the Visible and Uncovered Piece conditions as factors (Figure 4B, middle). Similar to the linear model, ANOVA results indicated that there was higher N1 amplitude for 32 visible pieces compared with 4 visible pieces, *F*(1, 19) = 30.80, *p* < .001, $\eta_{g}^{2}=.08$, whereas the N1 amplitudes among the uncovered pieces were comparable, *F*(7, 133) = 0.69, *p* = .68, $\eta_{g}^{2}=.01$. Moreover, there was a significant interaction between Visible and Uncovered Pieces, *F*(7, 133) = 2.20, *p* = .038, $\eta_{g}^{2}=.02$. The significant interaction was resolved by testing the uncovered piece condition effects in separate one-way ANOVAs for the two visible piece conditions: There was a main effect of Uncovered Pieces for 32 visible pieces, *F*(7, 133) = 2.27, *p* = .033, $\eta_{g}^{2}=.04$ but no significant effect of Uncovered Pieces for 4 visible pieces, *F*(7, 133) = 0.67, *p* = .69, $\eta_{g}^{2}=.01$. For 32 visible pieces, we tested each uncovered piece condition by running separate pairwise *t*-test comparisons. The results revealed that the condition with the largest number of uncovered pieces (32 pieces) significantly differed from any other condition: 0 piece, *t*(19) = −3.71, *p* = .002, *d* = −0.83; 1 piece, *t*(19) = −2.69, *p* = .015, *d* = −0.60; 2 pieces, *t*(19) = −3.49, *p* = .002, *d* = −0.78; 4 pieces, *t*(19) = −2.22, *p* = .038, *d* = −0.50; 28 pieces, *t*(19) = −2.26, *p* = .036, *d* = −0.51; 30 pieces, *t*(19) = −3.04, *p* = .007, *d* = −0.68; and 31 pieces, *t*(19) = −3.94, *p* = .001, *d* = −0.88. None of the other comparisons were significant, *t*s(19) < 1.03, *p*s > .32, *d*s < 0.23.

#### P3

Figure 4A (right) illustrates the mean P3 amplitude during the uncovered scene phase for different uncovered scenes. The mean P3 amplitude was 2.20 (±0.35) μV for 4 visible pieces and 1.44 (±0.36) μV for 32 visible pieces. A multiple linear regression model on the P3 amplitude showed that there was a significant intercept (β = 1.74, CI [1.78, 2.30], *t*(316) = 6.08, *p* < .001), indicating that the expected P3 amplitude for 4 visible pieces would have been 1.74 μV when the number of total game pieces would have been zero. The expected P3 intercept for the 32 visible pieces was comparable to 4 visible pieces (β = 0.41, CI [−0.86, 1.69], *t*(316) = 0.63, *p* = .53). There was a significant effect of total game pieces seen on the board for 4 visible pieces (β = 0.02, CI [0.00, 0.05], *t*(316) = 2.01, *p* = .045). The slope for 32 visible pieces significantly differed from the 4 visible pieces (β = −0.04, CI [−0.07, −0.01], *t*(316) = −2.32, *p* = .021), reflecting a negative relationship between the linear fits of the 4 and 32 visible pieces.

The P3 results showed that there was a linear relationship between the P3 amplitude and its predictor, the total game pieces. To examine how uncovered game pieces qualitatively affect P3, we further analyzed the data using a two-way repeated-measures ANOVA on the P3 mean amplitude, with the Visible and Uncovered Piece conditions as factors (Figure 4B, right). Similar to the linear model, ANOVA results indicated that there was higher P3 amplitude for 4 visible pieces compared with 32 visible pieces, *F*(1, 19) = 15.85, *p* < .001, $\eta_{g}^{2}=.03$. Moreover, there was a significant effect of Uncovered Pieces, *F*(7, 133) = 2.69, *p* = .012, $\eta_{g}^{2}=.03$, and a significant interaction between Visible and Uncovered Pieces, *F*(7, 133) = 2.84, *p* = .009, $\eta_{g}^{2}=.03$. The significant interaction was resolved by testing the uncovered piece condition effects in separate one-way ANOVAs for the two visible piece conditions: There was a main effect of Uncovered Pieces for 4 visible pieces, *F*(7, 133) = 3.53, *p* = .002, $\eta_{g}^{2}=.08$, but no significant effect of Uncovered Pieces for 32 visible pieces, *F*(7, 133) = 1.99, *p* = .062, $\eta_{g}^{2}=.04$. For 4 visible pieces, we tested each uncovered piece condition by running separate pairwise *t*-test comparisons. The results revealed that the condition with the smallest number of uncovered pieces (0 pieces) significantly differed from any other condition: 1 piece, *t*(19) = −3.12, *p* = .015, *d* = −0.60; 2 pieces, *t*(19) = −3.65, *p* = .002, *d* = −0.82; 4 pieces, *t*(19) = −3.25, *p* = .004, *d* = −0.73; 28 pieces, *t*(19) = −3.13, *p* = .006, *d* = −0.70; 30 pieces, *t*(19) = −4.15, *p* = .001, *d* = −0.93; 31 pieces, *t*(19) = −3.15, *p* = .005, *d* = −0.70; and 32 pieces, *t*(19) = −3.12, *p* = .006, *d* = −0.70. None of the other comparisons were significant, *t*s(19) < 1.50, *p*s > .15, *d*s < 0.34.

## Discussion

We examined the underlying mechanisms of the (under) estimation of the number of hidden objects in partially occluded scenes. Observers viewed a game board that was initially partially occluded before it was uncovered, while we measured ERPs that are linked to early and high-level visual processing stages. We found that the P1 amplitude increased with numerosity during the partially occluded and the uncovered phases, indicating that P1 is sensitive to continuous visual cues. Moreover, the N1 amplitude increased with the number of initially visible game pieces in the partially occluded phase. Importantly, its activity was not modulated by the number of game pieces being uncovered, but it was significantly higher in the completely filled game board after being uncovered. This suggests that the N1 component is sensitive to the overall Gestalt structure. We also found that the P3 activity had a positive relationship with the number of game pieces uncovered for 4 visible pieces and, in contrast, a negative relationship with the number of game pieces for 32 visible pieces. This result is likely to reflect individuals*’* different expectations for the two partially visible scenes. These observations go against the constant density prediction that would assume the same density and number of objects in the equal-sized visible and hidden areas. They are, however, in line with recent behavioral findings indicating that observers underestimate the number of hidden objects in partially occluded scenes (Men et al., 2023). Our results suggest that the observers did not expect to see additional pieces for a partially filled scene but they expected the scene to be completely filled if the initially visible area was completely filled.

Our findings align with studies showing that perceptual completion recruits high-level processes, rather than being a result of sole stimulus-driven processes (de Wit & van Lier, 2002; van Lier, van der Helm, & Leeuwenberg, 1995; Sekuler, 1994). What determines filled-in or completed representation in partially occluded objects or scenes is generally a challenging question: The relation between what is actually perceived and what is believed to be seen, that is, between the perceptual (bottom–up) and recognition (top–down) processes, respectively, is still unclear (Kellman, 2001). At the object level, on the one hand, Gestalt principles suggested that amodal completion is an outcome of bottom–up factors such as proximity, similarity, symmetry, and continuation, and completion is relatively insensitive to high-level processes such as beliefs and expectations (Wouterlood & Boselie, 1992; Kellman & Shipley, 1991; Kanizsa, 1979, 1985). On the other hand, earlier research indicated that some other forms of amodal completion are driven by top–down factors (Nanay, 2022; Vrins, de Wit, & van Lier, 2009; van Lier, van der Helm, & Leeuwenberg, 1994), such as global visual context (Rauschenberger et al., 2004), previous experiences (Plomp & van Leeuwen, 2006; Joseph & Nakayama, 1999), and familiarity or knowledge of a given object (Yun, Hazenberg, & van Lier, 2018; Hazenberg, Jongsma, Koning, & van Lier, 2014). In amodal completion, observers are typically aware of occluders and the fact that parts of the scene are hidden from view. Similarly, at the level of scene perception, we expected that inferring information from hidden areas would be accompanied by higher level processes. As predicted, there was a relatively higher influence of high-level processing stages in response to partially occluded scenes. We found that early visual ERPs, P1 and N1, were not modulated by incompatibility between the partially occluded and uncovered scenes. Instead, our results showed that P3 varied with the number of items revealed behind the occluder, linearly growing for a small number of initially visible pieces and, conversely, linearly decreasing for a large number. The P3 component, along with several other ERPs such as MMN, feedback-related negativity, or N400, is associated with expectation violation (Stefanics & Czigler, 2015; Holroyd et al., 2002; Friedman et al., 2001; Miltner et al., 1997), but frequently is linked with surprise and conflict with one*’*s expectancies: The higher the conflict with expectations, the higher the amplitude (Mars et al., 2008; Polich, 2007; Friedman et al., 2001; Polich & Kok, 1995). The lowest P3 amplitude was observed when zero game pieces were revealed for the 4 visible pieces, or when 32 pieces were revealed for the 32 visible pieces. These results suggest that individuals had an expectation of seeing no game pieces behind the occluder when the partially covered scene had only a few items visible, but they expected a fully covered board when the initially visible area was completely filled. This finding is particularly relevant to perceptual illusion of absence showing that the visual system can create an illusion of absence for hidden areas by relying on visible information and contextual cues (Svalebjørg, Øhrn, & Ekroll, 2020; Øhrn, Svalebjørg, Andersen, Ring, & Ekroll, 2019). For example, Øhrn, et al., 2019 found that the illusion of absence is stronger for narrower occluders, suggesting that hidden area estimation can be affected by the visible information such as occluder size. In line with this, our results indicate that fewer visible items lead to a stronger illusion of absence.

For the stage of early visual processing, our findings corroborate previous studies indicating that P1 amplitude is modulated by visual features (Proverbio & Zani, 2002; Johannes et al., 1995). We found that P1 correlated with the number of items being revealed behind the occluder irrespective of the number of initially visible items. This was also evident in the partially occluded scene presentation with higher P1 amplitudes for the 32 initially visible pieces compared with 4 initially visible pieces, suggesting that there is a link between the P1 amplitude and continuous visual properties. Although numerosity is considered a primary visual feature (Anobile et al., 2016), it is not completely independent of other continuous visual cues such as aggregate surface, convex hull (contour around an array of items), and density (aggregate surface divided by convex hull; Gebuis & Reynvoet, 2012, 2013). Therefore, it is possible that P1 is sensitive to continuous visual cues rather than numerosity. Furthermore, the manipulation of numerosity involved a change in luminance in our study: The scenes with more game pieces had lower luminance compared with scenes with fewer game pieces. Given that the P1 amplitude did not increase with higher luminance in a given scene, contradicting its previous interpretation (Proverbio & Zani, 2002; Johannes et al., 1995), we assume that the P1 activity was not primarily driven by luminance in our study. Another interpretation of the P1 component is to index suppression in the sensory gain control mechanism (Hillyard, Vogel, & Luck, 1998; Luck & Hillyard, 1994) and inhibitory processes in selective attention (Slagter, Prinssen, Reteig, & Mazaheri, 2016). Following these accounts, in our study, increased P1 would imply suppression of irrelevant stimuli; hence, more items revealed behind the occluder could be interpreted as a higher attentional suppression. Future studies may look into the distinction between the influences of sensory cues and attention on the P1 activity.

In addition, we found an increased N1 amplitude for scenes that were completely filled with game pieces. This aligns with previous studies showing that illusory contours are linked to a negative deflection at parietal and occipital regions around 150–180 msec poststimulus (Proverbio & Zani, 2002; Korshunova, 1999; Sugawara & Morotomi, 1991). Crucially, we did not observe a scaling of N1 with scene conditions with differing numerosities, suggesting that N1 is associated with the overall Gestalt structure of a scene, rather than continuous visual cues. Earlier studies reported that amodal completion occurs in early visual areas (Erlikhman & Caplovitz, 2017; Lee & Nguyen, 2001; for a review, see Thielen, Bosch, van Leeuwen, van Gerven, & van Lier, 2019), and this was supported by a number of EEG studies, showing that Gestalt visual stimuli lead to an increased N1 amplitude (Marini & Marzi, 2016; Conci et al., 2011; Brodeur, Lepore, & Debruille, 2006; Pegna, Khateb, Murray, Landis, & Michel, 2002; Herrmann & Bosch, 2001). Extending these findings on amodal completion at the object level, we showed that N1 also indexes perceptual completion that is compatible with Gestalt structure at the level of scenes.

Together, our findings present electrophysiological evidence that high-level processes affect perceptual completion in complex scenes. Combining the P1 and N1 results, we did not find bottom–up influences on partially covered scene presentations, whereas our P3 findings indicated that the completions of partially occluded scenes were defined by top–down processes. Our study showed that the inferences about numerosity in hidden objects is driven by high-level processes such as expectation.

## Data Availability

The data supporting the findings of this study and the statistical analysis code used in the article are available at https://doi.org/10.18112/openneuro.ds005586.v2.0.0.
